# Slowed epigenetic aging in Olympic champions compared to non-champions

**DOI:** 10.1007/s11357-024-01440-5

**Published:** 2024-11-27

**Authors:** Zsolt Radák, Dóra Aczél, Iván Fejes, Soroosh Mozaffaritabar, Gabor Pavlik, Zsolt Komka, László Balogh, Zsofia Babszki, Gergely Babszki, Erika Koltai, Kristen M. McGreevy, Juozas Gordevicius, Steve Horvath, Csaba Kerepesi

**Affiliations:** 1 Hungarian University of Sport Science, Budapest, Hungary; 2 University of Pécs, Pécs, Hungary; 3 Sapientia University, Sfântu Gheorghe, Romania; 4 Waseda University, Tokorozawa, 2-579-15 Japan; 5 Institute for Computer Science and Control (SZTAKI), Hungarian Research Network (HUN-REN), Budapest, Hungary; 6 University of Debrecen, Debrecen, Hungary; 7 Department of Biostatistics, Fielding School of Public Health, University of California Los Angeles, Los Angeles, CA 90095 USA; 8 Epigenetic Clock Development Foundation, Torrance, CA USA; 9 Altos Labs, Cambridge Institute of Science, Cambridge, UK; 10 Department of Information Systems, Eötvös Loránd University, Budapest, Hungary

**Keywords:** Epigenetic aging, Olympic champions, Epigenetic clocks

## Abstract

The lifestyle patterns of top athletes are highly disciplined, featuring strict exercise regimens, nutrition plans, and mental preparation, often beginning at a young age. Recently, it was shown that physically active individuals exhibit slowed epigenetic aging and better age-related outcomes. Here, we investigate whether the extreme intensity of physical activity of Olympic champions still has a beneficial effect on epigenetic aging. To test this hypothesis, we examined the epigenetic aging of 59 Hungarian Olympic champions and of the 332 control subjects, 205 were master rowers. We observed that Olympic champions exhibit slower epigenetic aging, applying seven state-of-the-art epigenetic aging clocks. Additionally, male champions who won any medal within the last 10 years showed slower epigenetic aging compared to other male champions, while female champions exhibited the opposite trend. We also found that wrestlers had higher age acceleration compared to gymnasts, fencers, and water polo players. We identified the top 20 genes that showed the most remarkable difference in promoter methylation between Olympic champions and non-champions. The hypo-methylated genes are involved in synaptic health, glycosylation, metal ion membrane transfer, and force generation*.* Most of the hyper-methylated genes were associated with cancer promotion. The data suggest that rigorous and long-term exercise from adolescence to adulthood has beneficial effects on epigenetic aging.

**Supplementary Information:**

The online version contains supplementary material available at 10.1007/s11357-024-01440-5.

## Introduction

The 2024 Summer Olympic Games in Paris featured over 10,000 athletes from around the world, drawing significant public attention. These athletes train between 2 and 10 h per day, 5–6 times a week, far exceeding the suggested guidelines for health benefits [1].

Competitive sports focus on achieving peak performance rather than promoting health, yet athletes can reap benefits from a dose-dependent relationship to physical activity. Strenuous exercise has been associated with health risks [2], including cardiac and orthopedic overuse among elite athletes [3]; however, moderate activity and elite athletes still can have health benefits. For example, every exercise load between moderate to vigorous activity suppresses mortality from cardiovascular diseases compared to those who are physically inactive [4]. Other studies have shown that elite athletes, such as the first 20 runners to break the 4-min mile and Finnish male elite athletes, enjoy increased lifespans and lower mortality rates [5, 6]. Similarly, Tour de France cyclists exhibit greater longevity compared to the general population [7]. However, German athletes participating multiple times in the Olympics had a lower survival rate, possibly due to the prolonged high levels of exercise-induced metabolism [8].

Furthermore, genetic factors contribute to success in elite sports and the body’s adaptation to training, including injury risk and disease prevention [9]. However, an assessment of 33 disease-related mutations and polymorphisms in Spanish elite athletes revealed that they are not genetically predisposed to lower disease risk compared to non-athletes [10]. Therefore while genetics does not explain longevity or health benefit differences between athletes and non-athletes, recent evidence points to exercise modulating the epigenome which can predict differential health and mortality risks between athletes and non-athletic counterparts [11].

One aspect of the epigenome is DNA methylation (DNAm), and DNAm-based aging clocks have been developed to provide insights into aging rates and mortality. The first-generation epigenetic clocks (e.g., Horvath’s pan-tissue clock, the blood-based Hannum clock, and the SkinBlood clock) predicted age accurately and exhibited associations with clinical biomarkers and mortality risk [12–14]. Second-generation epigenetic clocks (e.g., PhenoAge, GrimAge, and DunedinPACE) showed even stronger associations with mortality risk and some age-related conditions [15–17]. Recently, we developed DNAmFitAge, which is a biological age indicator incorporating physical fitness like VO2max and grip strength [18]. Physically active individuals exhibit younger DNAmFitAge and better age-related outcomes: lower mortality risk, reduced coronary heart disease risk, and increased disease-free status [11].

Hungary ranks 8th in the all-time Summer Olympic Games medal count, with 181 gold medals, despite a population of less than 10 million. In this study, blood samples and lifestyle data were collected from 59 Hungarian Olympic gold medalists. Many potential champions begin training at a young age (6–10 years), which may have long-term epigenetic consequences. Adverse childhood experiences are known to alter DNA methylation patterns and accelerate epigenetic aging, suggesting that intense exercise during childhood could also impact DNA methylation-based aging [19].

Here, we apply DNAm-based aging clocks to investigate the epigenetic aging of Olympic gold champions, compared to non-champions.

## Methods

### Study participants

Fifty-nine Olympic champions (*N* = 10 female and *N* = 49 male) and 329 control (*N* = 161 female and *N* = 168 male) subjects voluntarily participated in this study, which was approved by the National Center for Public Health (7147–6/2022EUIG). Olympic champions from fencing, soccer, gymnastics, kayak-canoe, modern pentathlon, swimming, wrestling, water polo, and short-track skating completed a questionnaire regarding their health, educational status, and lifestyle, including exercise habits. Of the 329 control subjects, 205 were master rowers who participated in the World Rowing Masters Regatta in Velence, Hungary, and healthy untrained volunteers. Blood samples were collected and stored in evacuated tubes containing EDTA as an anticoagulant for determination of erythrogram. Blood samples were centrifuged and stored at − 80 °C. Whole blood samples were used to isolate DNA for methylation.

### Measurement of DNA methylation

In this study, we analyzed four methylation batches (we referred to them as MET2019, MET2020, MET2022, and MET2023, Table S1). MET2019 batch contained 263 control samples that were used in our previous study and measured by the Infinium MethylationEPIC (850 k) BeadChip (Illumina Inc., San Diego, CA) according to the manufacturer’s protocol as described earlier [11]. The 33 control samples of batch MET2020 and the 59 Olympic champion samples of batch MET2022 were measured by using the Infinium MethylationEPIC BeadChip, while the 33 control samples of batch MET2023 were measured by the Infinium MethylationEPIC v2.0 BeadChip.

### Epigenetic biomarkers

Epigenetic clocks were applied using the DNA Methylation Age Calculator of the Clock Foundation Team (https://dnamage.clockfoundation.org/clock). We applied the Horvath pan-tissue clock [12], the blood-based Hannum clock [14], SkinBlood clock [13], PhenoAge clock [15], DNAmFitAge clock [18], and GrimAge v1 and v2 clocks [16, 20]. We calculated age acceleration as the residual, per sample, after fitting the predicted age to chronological age (i.e., the age acceleration is the deviation from the trend). The telomere length was also evaluated by using the DNA Methylation Age Calculator (https://dnamage.clockfoundation.org), which estimated the telomere length from methylation data [21].

### Normalized beta values

Raw methylation signal intensities were retrieved using the function read.metharray.exp of the minfi v1.40.0 R package, followed by linear dye bias correction and noob background correction to account for technical variation in background fluorescence signal [22]. Specifically, the β-value was calculated from the intensity of the methylated and unmethylated sites, as the ratio of fluorescent signals. The Clock Foundation and other predictor algorithms initially employed the standard EPIC or 450 K/27 K Illumina array probe naming convention for constructing all clocks. However, a significant drawback of the EPICv2 array emerged with the removal of over 7000 critical probes. To address this challenge, we devised generalized linear models algorithms capable of predicting missing probes by leveraging the information gleaned from existing EPICv2 probes.

### Differently methylated promoter analysis

For the differently methylated promoter analysis, we merged the normalized beta values of the batch MET2019, MET2020, MET2022, and MET2023. As the four batches were measured by two different methylation arrays (EPICv1 and EPICv2), we used only the common CpG sites (729,536 probes). We replaced the incorrect beta values (< 0 or > 1) with the mean value of the given CpG site. Then, the differently methylated promoter analysis was performed by using generalized linear models with binomial link function. For each gene, the predictors (independent variables) were the age and the mean beta value of the promoter region, while the dependent (response) variable was a binary variable with a value of 1 for Olympic champions and 0 for the controls. We considered a CpG site to be located in a promoter region if it was annotated as TSS1500, TSS200, 5′UTR, or 1stExon [23]. We used FDR correction for the *p*-values.

### Statistical analysis

Python packages were used for statistical analysis (numpy = 1.24.4, pandas = 2.0.3, scipy = 1.11.1, statannot = 0.2.3, statsmodels = 0.14.2). We used two-sided independent Student’s *t*-tests to compare two groups. If *p* values were indicated by an asterisk, we used the notations as follows: ns, *p* > 0.05; *, 0.01 < *p* ≤ 0.05; **, 0.001 < *p* ≤ 0.01; ***, *p* ≤ 0.001; and ****, *p* ≤ 0.0001. We did not correct *p*-values for multiple-test comparisons, except for the differently methylated gene promoter analysis (FDR).

## Results

### Slowed epigenetic aging of Olympic champions compared to non-champions

**Fig. 1:**
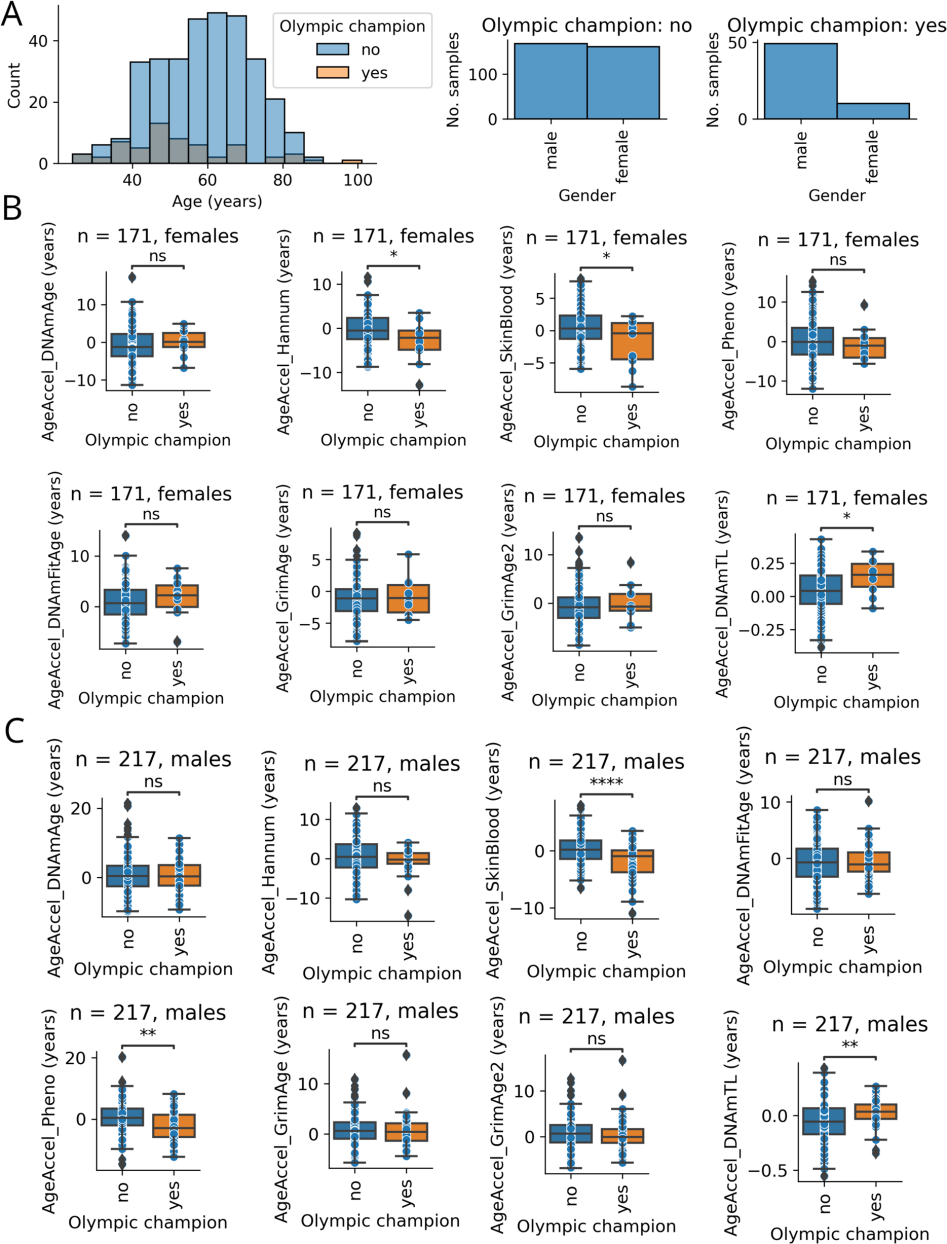
Slowed epigenetic aging of Olympic champions compared to non-champions*.* **A** The age and gender distribution of the Olympic champions and the controls (non-champions). **B** Epigenetic age accelerations among female Olympic champions calculated by seven epigenetic clocks. The age-adjusted residual of DNA methylation-based telomere length predictions (AgeAccel_DNAmTL) are also presented. **C** A similar analysis for males

We measured the epigenetic age and the DNAm telomere length of 59 Olympic champions (*N* = 10 female and *N* = 49 male) and 332 control (*N* = 161 females and *N* = 171 males) with an age range between 24 and 101 years (Fig. 1A). The female Olympic champions’ mean age was 53.3 ± 22.3 and the male Olympic champions had a mean of 52.4 ± 14.9 years. The female non-champions’ mean age was 60.3 ± 11.8, while the male non-champions had a mean of 58.3 ± 13.4. The Hannum and SkinBlood clocks showed significantly decreased epigenetic age acceleration (i.e., age-adjusted age prediction) for female champions compared to female non-champions (Figs. 1B and S1A), while the SkinBlood and PhenoAge clocks showed significantly decreased age acceleration for male champions compared to male non-champions (Figs. 1C and S1B). We also predicted the telomere length from the methylation data and found that the age-adjusted DNAm telomere length increased in Olympic champions compared to the non-champions for both sexes (Figs. 1B and C and S1AB).

### For males, recent medalists aged slower compared to past medalists, while the opposite effect was observed for females

**Fig. 2:**
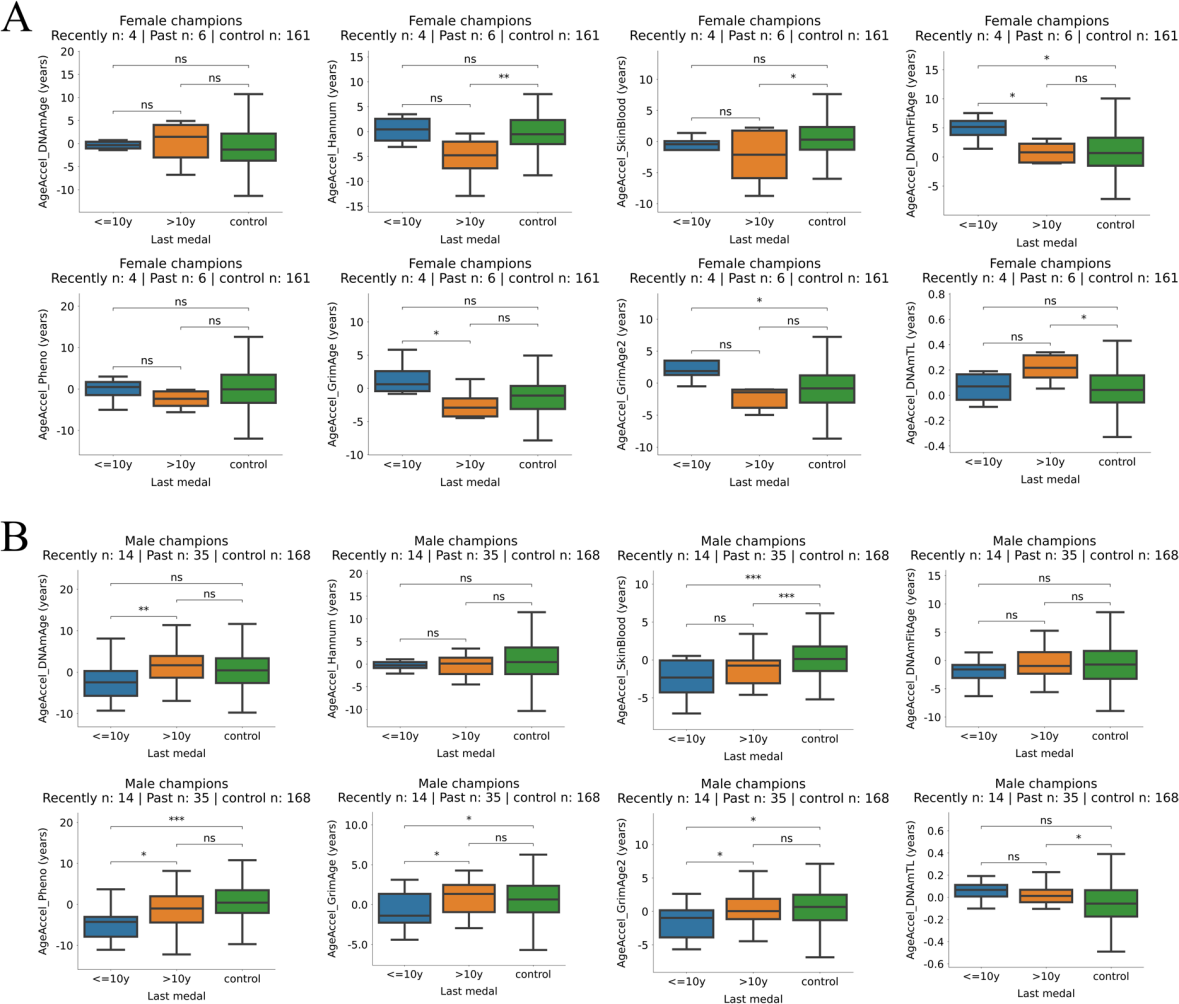
For males, recent champions age slower compared to past champions while the opposite effect is true for females. **A** Epigenetic age accelerations and DNAmTL in female Olympic champions who earned championship medals in the last 10 years compared to the rest of female champions and non-champions. **B** Similar analysis as in **A** but for males

We calculated the time elapsed between the date of sampling and the date of the last championship medal won by a given individual of the Olympic champion cohort. We compared the epigenetic age acceleration between those Olympic champions who earned any medal in Olympic games, World, European, or League Championships less than 10 years before blood sampling (“recent medalists”) and more than 10 years before blood sampling (“past medalist”). For female Olympic champions, DNAmFitAge and GrimAge showed significantly higher epigenetic age acceleration for recent medalists compared to past medalists. On the other hand, for male champions, DNAmAge, DNAmPhenoAge, GrimAge, and GrimAge2 clocks showed significantly lower epigenetic age acceleration for recent medalists compared to past medalists (Fig. 2).

### Among Olympic champions, wrestling had higher epigenetic age acceleration compared to gymnastics, fencing, and water polo

**Fig. 3:**
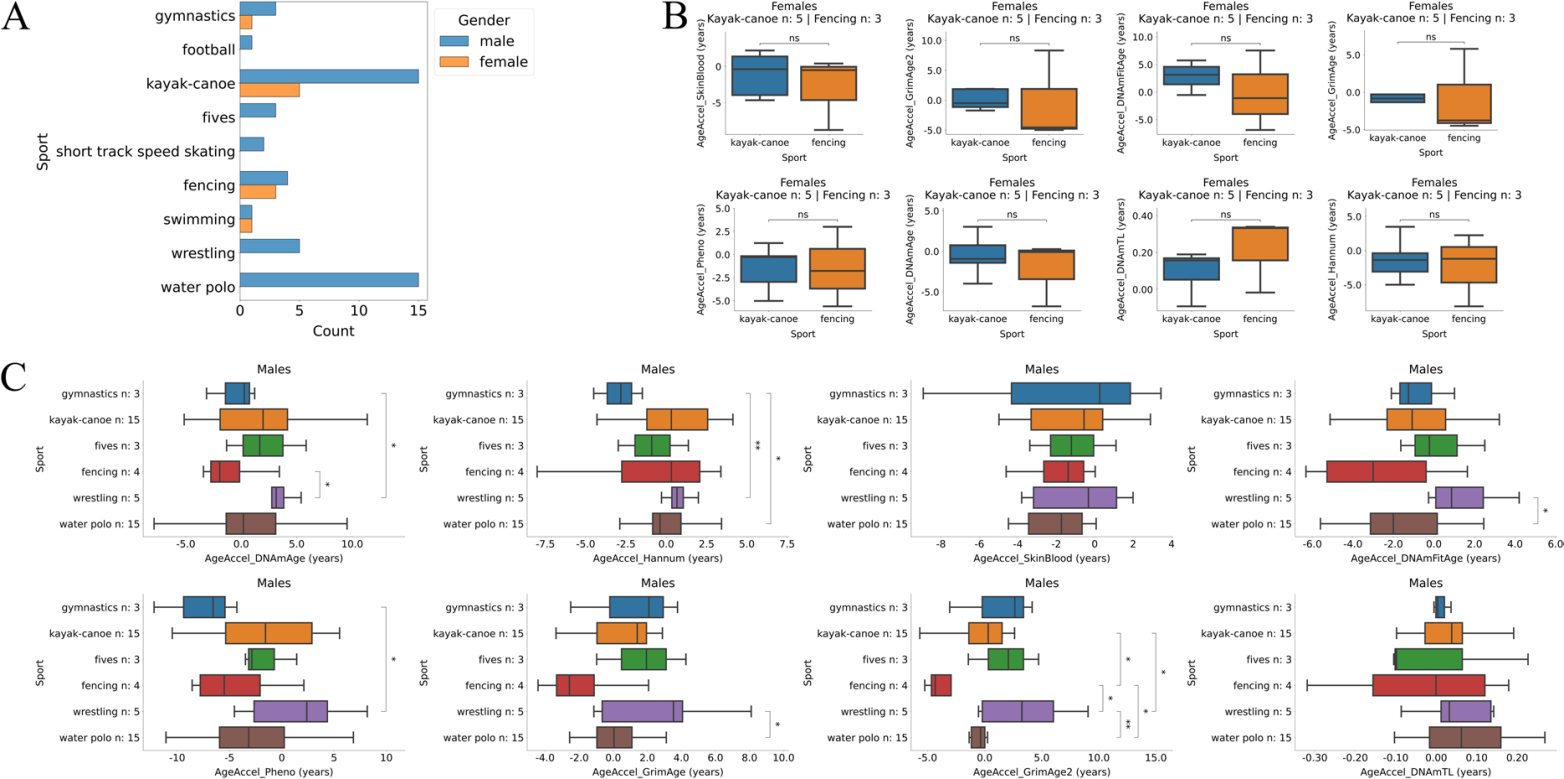
Among Olympic champions, wrestling had higher epigenetic age acceleration compared to gymnastics, fencing, and water polo. **A** Distribution of sports of the 59 Olympic champions. **B** Sport-specific age acceleration of female champions. Only the sports with at least three female samples were considered. **C** A similar analysis for males

Despite the small number of subjects in each sport, we compared age accelerations in different sports. Only those sports were compared where the number of samples was greater than three (Fig. 3A). We did not find any significant differences among female Olympic champions (Fig. 3B). However, the age acceleration of male Olympic champions in wrestling was significantly higher compared to that of gymnastics, fencing, and water polo according to some epigenetic aging clocks (Fig. 3C).

### Differently methylated gene promoters in Olympic champions compared to non-champions

**Fig. 4:**
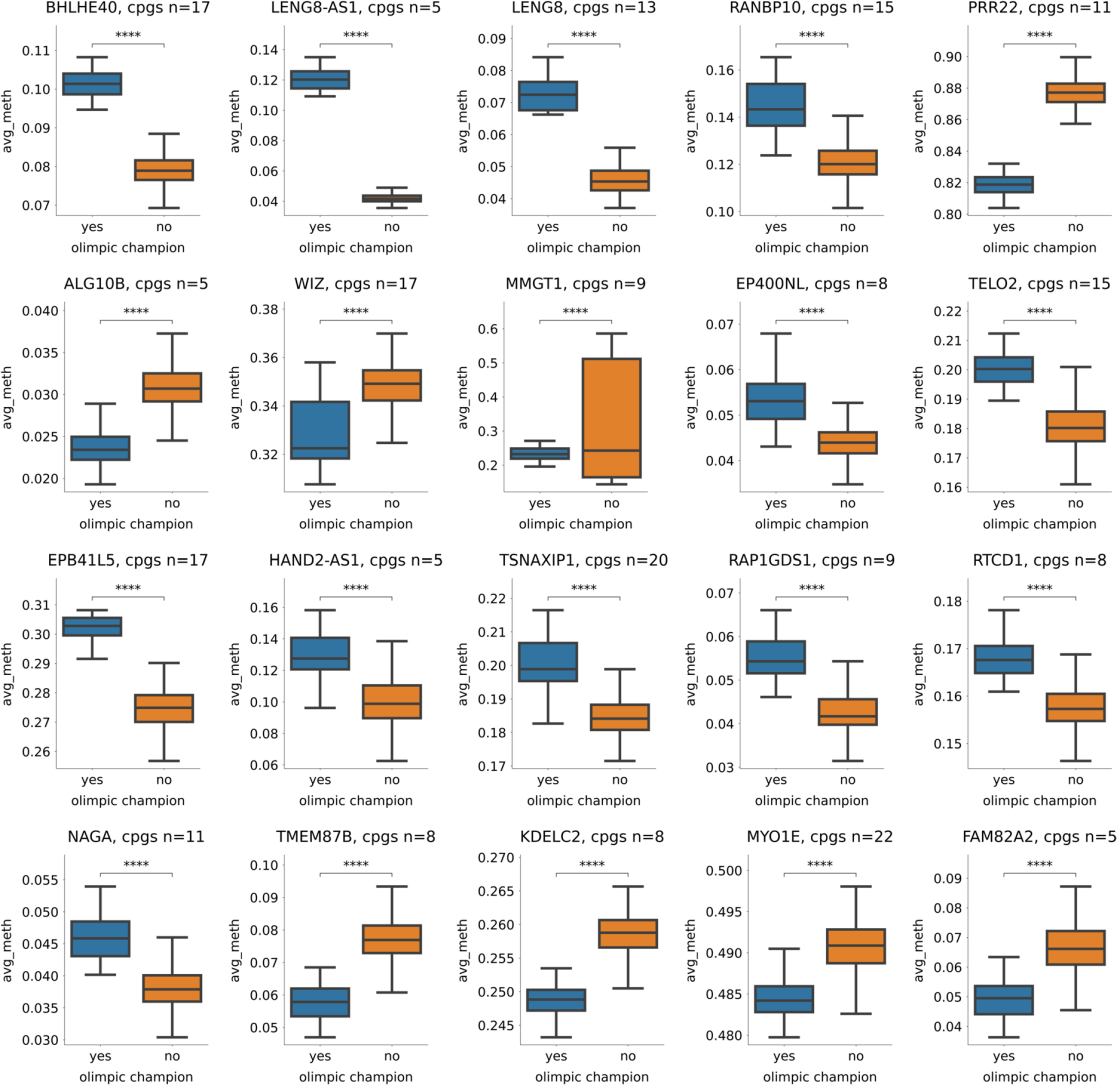
Top 20 differently methylated gene promoters comparing Olympic champions and non-champions (FDR corrected *p*-value < 2.24e-11 for each gene promoter). Probe name, chromosome, strand (− or +), genomic position, genomic features, regulatory feature group, and gene names are indicated for each CpG site

For each human gene, we calculated the mean methylation levels of the CpG sites associated with the promoter region (Supplementary Table S2). We further examined the top 20 differently methylated genes (FDR corrected *p*-value < 2.24e-11) between Olympic champions and non-champions (Fig. 4). Among these genes, the following genes showed hypo-methylation in Olympic champions*: PRR22*, *ALG10B*, *WIZ*, *MMGT1*, *TMEM87B*, *KDELC2*, *MYO1E*, and *FAM82A2.* The most remarkable hyper-methylated genes of Olympic champions included *BHLH40*, *LENG8-AS1*, *LENG8*, *RANBP10*, *EP400NL*, *TELO2*, *EPB45L5*, *HAND2-AS1*, *TSNAXIP1*, *RAP1GDS1*, *RTCD1*, and* NAGA.*

Gene enrichment analysis revealed the overrepresentation of IL-2/STAT5 signaling (MYO1E, GLIPR2, PUS1, BHLHE40, SLC2A3, and FAH) and mTORC1 signaling (BTG2, PSMC6, BHLHE40, SC5D, and SLC2A3) genes among the top 100 differently methylated genes between Olympic champions and non-champions (adjusted *p*-value: 0.01217 and 0.04138, respectively, based on the MSigDB Hallmark 2020 library of the Enrichr web tool [24]).

## Discussion

This could be one of the first investigations on the aging process of every selected elite athletes, the Olympic gold medalists. DNA methylation-based aging clocks aimed to measure the rate of aging, which is significantly affected by lifestyle factors, like physical exercise. Most of the previous studies observed that elite athletes have longevity [5–7, 25, 26]. In line with this, here we additionally showed that Olympic champions tend to have a slower aging process by the DNA methylation-based aging clocks than non-champions. A slower epigenetic age not only predicted a decrease in mortality but also showed a decreased incidence of a variety of diseases [15]. To our knowledge, only one paper assessed the DNA methylation associated with aging of athletes showing that young athletes (mean age = 24.1 years) have accelerated aging assessed by the 5-CpG model compared to sedentary subjects [27]. However, this age acceleration was associated with the modified methylation of *TRIM59* and *KLF14* genes, which had health-promoting anti-tumor and anti-inflammatory effects. Multiline evidence suggests adolescent lifestyle factors could have long-term consequences via DNA methylation in adulthood [28]. Indeed, the results of the longitudinal FinnTwin12 study (*n* = 5114), which assessed lifestyle-related factors, reported that an unhealthy lifestyle during pubertal years is associated with accelerated biological aging in young adulthood [29]. Our data suggest that physical exercise even with big loads during adolescence (most of our champions had more than five training sessions at the age of 12) does not have negative consequences for DNA methylation-derived age, contrary to the champions showing decelerated aging.

The relationship between telomere length and physical activity is inconsistent. However, the meta-analysis of 43 studies suggests that regular aerobic exercise including vigorous intensity appears to help preserve telomere length [30]. Here, we report that champions have longer telomere lengths, calculated from DNA methylation data, than non-champions and this fits well with the data obtained on methylation-related aging.

It is well known that the adaptive response of different sports is dependent on the intensity and duration of exercise loads. Despite the small number of Olympic champions in each sport, we attempted to examine the sport-specific response to aging. Data suggests that wrestlers age faster than gymnasts, fencers, and water polo players. It is risky to generalize this finding because of the low number of subjects but the possible reasons behind this sport-specific difference could be due to different types of training, nutrition, and weight-controlling methods during the competitive periods, and education levels as well. In our cohort, fencers and water polo players were more highly educated than wrestlers.

Olympic champions showed the most remarkable hypo-methylation of the following genes*: PRR22*, *ALG10B*, *WIZ*, *MMGT1*, *TMEM87B*, *KDELC2, MYO1E*, and *FAM82A2.* Proline-rich transmembrane protein 2 is a protein that in humans is encoded by the *PRRT2* gene which seems to be crucial for synaptic health [31]. *ALG10B* gene-related proteins are important to glycosylation, while *MMGT1* regulates metal ion membrane transfer. WIZ gene regulates DNA transcriptional activity*. TMEM87B* has proposed roles in protein transport to and from the Golgi, as mechanosensitive ion channels, and in developmental signaling. *KDELC2* encoded proteins regulating NOTCH signaling. Myosin-Ie protein is coded by *MYO1E* gene; this myosin isoform, like others, uses the energy of ATP hydrolysis to interact with actin filaments to generate force, however in a greater degree than other myosin isoforms [32]. *FAM82A2* coded proteins that are involved in apoptosis [33] and differentiation in human muscle cells [34]. The genes which promoter regions were hypo-methylated in Olympic champions are regulating complex cellular signaling, transfer processes, differentiations, and force generation.

In the samples of Olympic champions, the following genes were hyper-methylated compared to non-champions: *BHLHE40*, *LENG8-AS1*, *LENG8*, *RANBP10*, *EP400NL*, *TELO2*, *EPB45L5*, *HAND2-AS1*, *TSNAXIP1*, *RAP1GDS1*, *RTCD1*, and *NAGA.* It has been shown that higher expression of *BHLHE40* might be involved in immunosuppression of pancreatic cancer [35]; therefore, it cannot be excluded that the modulation of this gene plays an important role in the exercise-induced immune response. Moreover, the methylation of *BHLHE40* gene negatively correlates with body mass index and related total energy and carbohydrate intake [36] which could fit to exercise-related increased metabolism. *RANBP10* codes proteins involved in prostate cancer [37] and glioblastoma [38]; therefore, silencing (i.e., hyper-methylation) of this gene could account for, at least a part, the cancer-suppressing effects of exercise. It has been shown that upregulation of *EP400NL* in lung adenocarcinoma tissue from cancer patients who have a smoking history [39] and EP400 interaction with c-Myc induce cancerous phenotypes [40]. *TELO2* gene encodes proteins that are involved in the regulation of telomere length and here we show that champions have longer predicted telomere length than non-champions. In addition to *TELO2*, TSNAXIP1-encoded proteins interact with SUN1 which is involved in the maintenance of telomere length [41] and *TSNAXIP-*coded proteins are crucial for spermatogenesis [42]. Association between protein 4.1, coded by *EPB41L3*, and actin suggests that this interaction contributes significantly to the stabilization of the spectrin-actin-protein 4.1 ternary complex and erythrocyte function [43]. HAND2-AS1 is a long non-coding RNA that is associated with the development of different tumors [44]. *RAP1GDS1* gene encodes guanine nucleotide exchange factor that regulates small GTPases, including RHOA, RAC1, and KRAS. *RAP1GDS1* plays a crucial role in cell signaling and DNA repair. *NAGA* gene encodes alpha-N-acetylgalactosaminidase, the enzyme that accumulates in the blood of tumor-bearing subjects [45]. The hyper-methylated (i.e., silenced) genes are generally involved in tumor suppression, telomere maintenance, fertility, and cellular signaling. The epigenetic adaptation of Olympic champions is confirmed by the meta-analysis of 165,000 former athletes showing reduced incidence and mortality in cardiovascular diseases and cancer among athletes compared to sedentary [46].

The present study investigated DNA methylation-associated aging of Olympic champions, which limited the number of subjects, but even with this limitation, to our knowledge, this is the only study that examined more than ten Olympic champions.

In summary, here, we investigated the DNAm epigenetic clocks and the DNAm telomere length in Olympic gold medalists. We explored the long-term epigenetic modifications that accompany elite sport, even after years have passed since the last training, and found that rigorous and long-term exercise from adolescence to adulthood has beneficial effects on epigenetic aging. Data revealed that the differently methylated promoters between Olympic champions and non-champions were associated with genes that were involved in the regulation of apoptosis, immune system, extra- and intra-cellular signaling, telomere maintenance, and cancer prevention. This study provides evidence that even top sport has health benefits, it decelerates the aging process and decreases the risk of a wide range of diseases.

## Supplementary Information

Below is the link to the electronic supplementary material.

Supplementary Table S1 Metadata of all samples and the results of the 7 epigenetic clock and the DNAm telomere length. (XLSX 98 KB)

Supplementary Table S2 Mean methylation levels of the CpG sites associated with the promoter region of each human gene, sorted by the age-adjusted and FDR corrected p-value of the age adjusted difference between Olympic champions and non-champions. (XLSX 3049 KB)

Supplementary Fig. S1 Predictions of the 7 epigenetic clocks and the DNAmTL for Olympic champions and non-champions. (**A**) For females, and (**B**) for males. Linear regression line (solid blue lines) of the predicted ages is also shown. The dashed orange line is the diameter (x=y). (PNG 1.30 MB)

High resolution image (TIFF 2122 KB)

## Data Availability

Raw and processed methylation data will be available on the GEO upon publication. We shared the metadata of all samples and the results of epigenetic clocks (Table S1).
