# Supplementary figures and images for: Slowed epigenetic aging in Olympic champions compared to non-champions
Source: GeroScience. 2024 Nov 27;47(2):2555–65. doi: 10.1007/s11357-024-01440-5 (PMC11978583; doi:10.1007/s11357-024-01440-5)

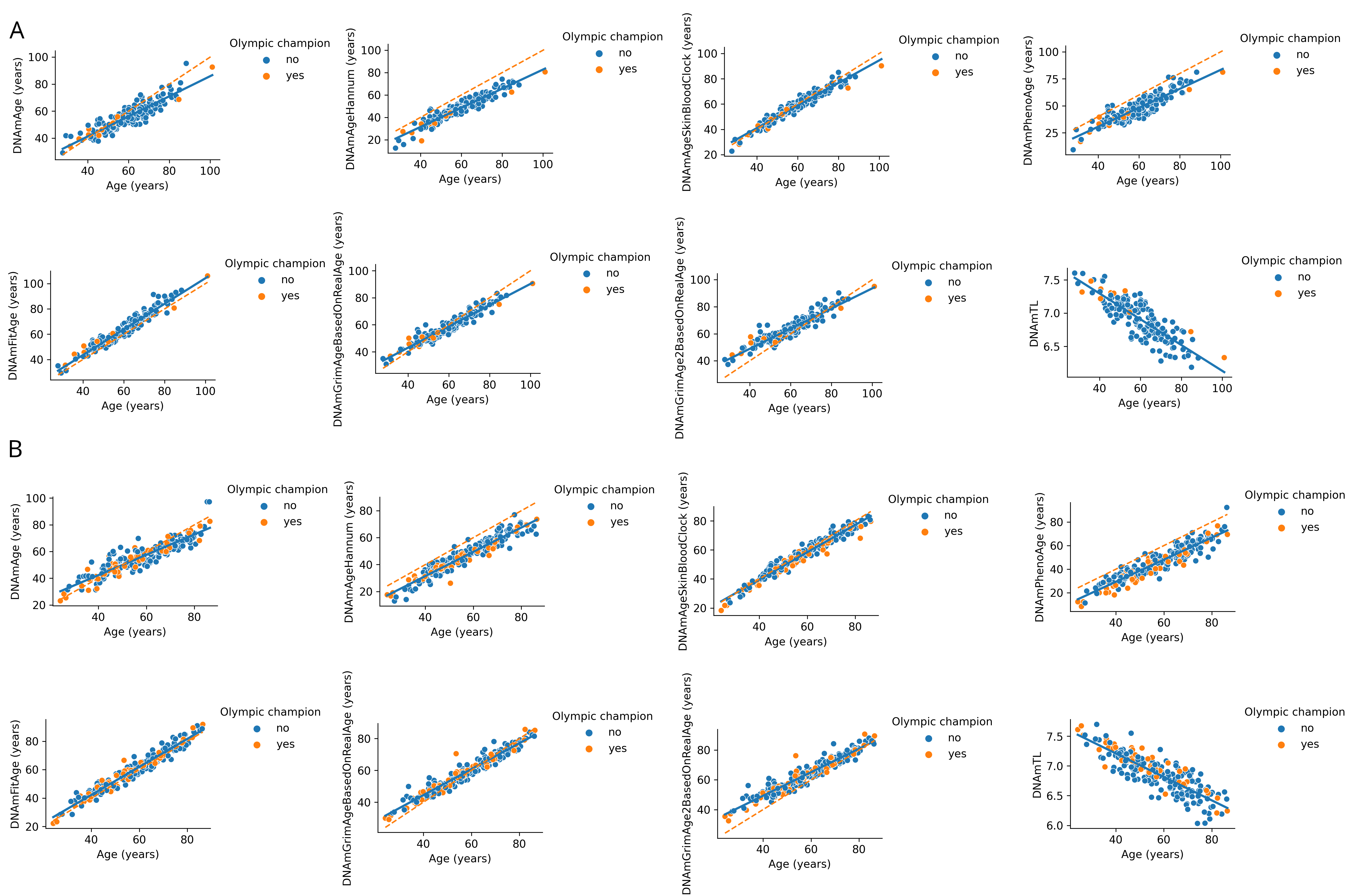

Supplement: Supplementary file 3 — Predictions of the 7 epigenetic clocks and the DNAmTL for Olympic champions and non-champions. (A) For females, and (B) for males. Linear regression line (solid blue lines) of the predicted ages is also shown. The dashed orange line is the diameter (x=y). (PNG 1.30 MB) [file 11357_2024_1440_Fig5_ESM.png]

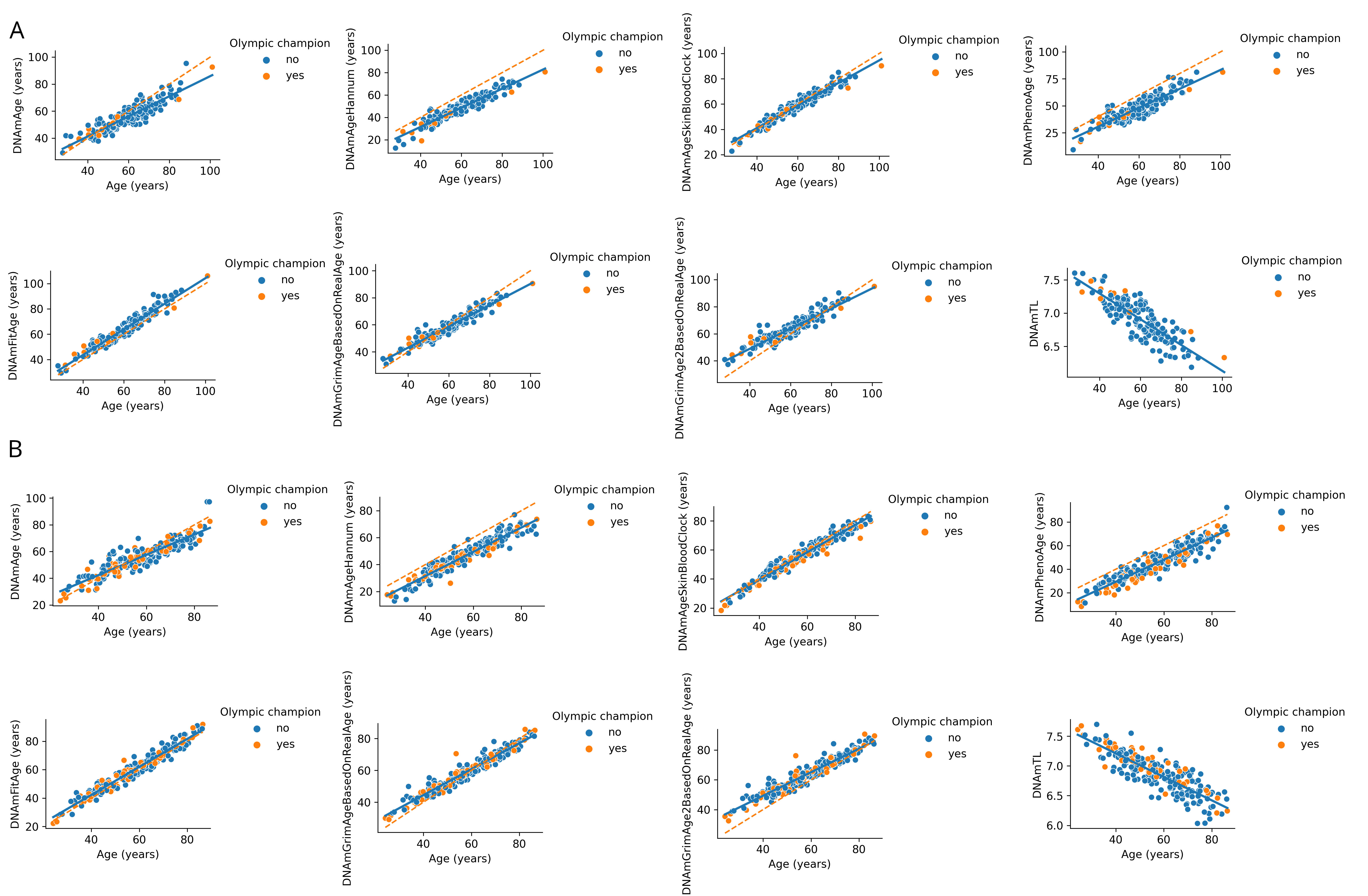

Supplement: Supplementary file 4 — High resolution image (TIFF 2122 KB) [file 11357_2024_1440_MOESM3_ESM.tiff]
